# Pharmacokinetic Studies in Elasmobranchs: Meloxicam Administered at 0.5 mg/kg Using Intravenous, Intramuscular, and Oral Routes to Nursehound Sharks (*Scyliorhinus stellaris*)

**DOI:** 10.3389/fvets.2022.845555

**Published:** 2022-03-23

**Authors:** Pablo Morón-Elorza, Carlos Rojo-Solís, Teresa Álvaro-Álvarez, Mónica Valls-Torres, Daniel García-Párraga, Teresa Encinas

**Affiliations:** ^1^Department of Pharmacology and Toxicology, Faculty of Veterinary Medicine, Complutense University of Madrid, Madrid, Spain; ^2^Fundación Oceanogràfic de la Comunitat Valenciana, Valencia, Spain; ^3^Veterinary Services, Oceanogràfic, Valencia, Spain

**Keywords:** meloxicam, shark, chondrichthyan, pharmacology, pharmacokinetics, non-steroidal anti-inflammatory drug (NSAID), half-life (T1/2)

## Abstract

Infectious and inflammatory diseases are the most frequently diagnosed pathologies in elasmobranchs maintained under human care. Non-steroidal anti-inflammatory drugs (NSAIDs) are frequently used in veterinary medicine for their anti-inflammatory, analgesic, and antipyretic properties. Meloxicam is a commonly prescribed NSAID in elasmobranchs, but there are still no published pharmacokinetic (PK) studies supporting its use in this group of animals. In this study, meloxicam was administered at a single dose of 0.5 mg/kg to eight healthy adult nursehound sharks (*Scyliorhinus stellaris*) intravenously (IV), intramuscularly (IM), and orally (PO), with a minimum 4-week washout period between administrations. Blood samples were obtained both beforehand and at predetermined times after each administration. Plasma concentrations were measured using a validated high performance liquid chromatography method, and PK data was obtained using a non-compartmental analysis. Meloxicam administered orally did not produce detectable concentrations in blood plasma, while mean peak plasma concentration was 0.38 ± 0.08 μg/ml after IM administration. The mean terminal half-life was 10.71 ± 2.77 h and 11.27 ± 3.96 h for IV and IM injections, respectively. The area under the curve extrapolated to infinity was 11.37 ± 2.29 h·μg/ml after IV injections and 5.98 ± 0.90 h·μg/ml after IM injections. Meloxicam administered IM had a mean absolute bioavailability of 56.22 ± 13.29%. These numbers support meloxicam as a promising drug to be used IM in nursehounds, questions the efficacy of its single PO use in elasmobranchs, elucidate the need for higher dosage regimes, and evidence the need for further PK studies in sharks and rays.

## Introduction

Aquariums serve as an indispensable research tool for the study of elasmobranchs, as by keeping them since 1860s, they have contributed to our knowledge base of sharks and rays in a wide variety of fields including, but not limited to, nutrition, reproduction, physiology, behavior, and pathology (1). They provide an easy and economical way for the scientific community to collect data on these animals that would otherwise be very difficult to obtain in the wild (2, 3). These contained facilities allow a 24 h monitoring of the study subjects, access to their medical history and data on their environmental parameters, which have all led to the identification of different pathologies in these species (2). Among these illnesses, infectious and inflammatory diseases have been the most frequently diagnosed in elasmobranchs (4). Disease management can be challenging, since the elasmobranch therapy database currently in use relies mostly on empirical data, and safe and effective therapeutic regimes for most antimicrobial and anti-inflammatory/analgesic agents in sharks and rays have not been yet established (5, 6). To this end, there is a need for updated and validated information regarding not only elasmobranch pathology, but also its accurate and effective clinical management.

The difficulty of evaluating pain in fish makes it challenging to properly manage pain and inflammation in elasmobranchs. Although it has been described that pain in fish produces stress, immunosuppression and behavioral changes, this group of animals lacks facial expression, live in water, and have social and behavioral responses very different to it when compared with mammals (7). Veterinary medicine addresses nociception from its origin to its transmission, modulation, and perception in the central nervous system. As part of a multimodal approach to analgesia, non-steroidal anti-inflammatory drugs (NSAIDs) are used to eliminate pain caused by inflammation, local anesthetics to stop pain transmission, and dissociative anesthetics and opioids to modulate cerebral pain perception. Furthermore, NSAIDs can have both central and peripheral effects since their mechanism of action is the inhibition of cyclooxygenase (COX) and thereby the production of prostaglandins (8, 9). Previous studies have confirmed the genetic expression of COX messenger ribonucleic acid in many fish species, thus prompting the use of NSAIDs as analgesics in these animals (10).

Pharmacokinetic (PK) studies require frequent handling of the subjects for blood collection, making them less suitable for easily stressed, large or dangerous species (5, 11). Very few PK studies have been carried out in elasmobranchs, and the ones performed used small benthic elasmobranch species such as the white bamboo shark (*Chiloscyllium plagiosum*) and the smooth dogfish (*Mustelus canis*) (12, 13). An advantage of using elasmobranchs for the development of PK studies is that they are susceptible to tonic immobility (TI) (11). TI is a form of physiological restraint commonly used while handling elasmobranchs for husbandry and medical procedures. It consists of a reflex that causes a temporary state of inactivity and mild sedation while the animal is placed in dorsal recumbency, reducing the need for anesthetics or sedative agents that could otherwise influence the PK parameters of the drug under study (14). Scyliorhinid sharks are frequently maintained in aquariums worldwide and are also frequently used as model species for elasmobranch studies (15, 16).

For this study, meloxicam, a NSAID that has a selective inhibition of the COX-2 enzyme, was chosen for its high importance and frequent use the clinical management of sharks and rays in aquariums (17, 18). The current recommended dosage for meloxicam can vary greatly among species, being lower in most domestic species and elasmobranchs (0.1-0.2 mg/kg), and higher in teleost fish (1 mg/kg), amphibians (1 mg/kg) and some avian species (1.0-1.6 mg/kg) (17, 19, 20). The popularity of meloxicam, along with the absence of PK data in elasmobranchs, called for the evaluation of its kinetics post- administration, which was achieved in this study using nursehounds (*Scyliorhinus stellaris*) as a model species.

## Materials and Methods

This experimental study was designed as an observational, prospective study. Animal handling and sample collection procedures were performed in agreement with and under the approval of the Animal Care and Welfare Committee at Oceanogràfic of Valencia and the Generalitat of València with the project reference ID OCE-22-19.

### Animals and Experimental Conditions

A group of eight adult nursehounds (four males and four females) under managed care at Oceanogràfic of Valencia (Valencia; Spain. http//www.cac.es/oceanografic) were used in this study. All sharks were determined healthy based on clinical history, physical examination, hematology, and plasma biochemistry results. On the first day of the study, weights were recorded for all the sharks, which ranged from 2.00 to 3.35 kg (mean ± SD was 2.69 ± 0.43 kg). Animals were classified as adults based on their total length measurements, which ranged from 81 to 91 cm (mean ± SD was 83.62 ± 3.20 cm), exceeding the average length of 77 cm in adult males and 79 cm in adult females (21). All animals have been kept at Oceanogràfic for at least 2 years. For the duration of the PK study, the sharks were temporarily transferred to 10,000-liter cylindrical tanks in the quarantine facility of the aquarium, with the following environmental parameters: 12:12 h artificial light: darkness periods, water temperature of 18°C and 34 g/L salt concentration; pH ranging from 7.9 to 8.1; zero ammonia and maximum 0.05 ppm nitrite and 50 ppm nitrate. Translocation of the animals to temporary tanks was performed more than 2 weeks prior to the onset of the study, so that all sharks were fully acclimated to their new environment. Animals were provided with shaded areas and environmental enrichment for resting and hiding. Sharks were fed previously thawed pieces of teleost and cephalopods once daily ad libitum, 6 days per week. Individuals were visually monitored throughout the study for possible clinical signs associated with handling, blood collection and drug toxicity.

### Experimental Protocol and Sampling

Meloxicam was administered at a single dose of 0.5 mg/kg to all eight nursehounds either by intravenous (IV), intramuscular (IM) or two different oral (PO) methods, with a minimum 4-week washout period between studies.

For parenteral administration, meloxicam (Metacam^®^ 5 mg/ml injectable solution, Boehringer Ingelheim S.A., Barcelona 08174, Spain) was delivered using a 23-gauge (0.6 x 25 mm) needle attached to a 1 ml syringe. Sharks were carefully captured with a rubber net and manually restrained underwater in a dorsoventral position, with the injection site above water for administration. IV administration was performed at the caudal vasculature using a lateral approach, and the administration was slow and constant, assuring that the vascular access was maintained throughout administration (5). IM injection was performed in the dorsal epaxial musculature; the needle was introduced approximately 20 mm into the musculature in the marginal region to the dorsal fin. Pressure was applied at the injection site after administration to minimize drug leakage (5).

For single dose oral administration, and to evaluate the two most common methods for oral drug delivery in sharks, two separate voluntary and forced administration studies were performed. In the first one, meloxicam tablets (Movalis^®^ 7.5 mg/tablet, Boehringer Ingelheim S.A., Barcelona 08174, Spain) were crushed to a powder and put into gelatin capsules, which were inserted into fish that were given to the sharks administered using a feeding stick, as the animals had been previously trained. Four individuals were medicated using herring and the remaining four using mackerel pieces. Animals voluntarily ate the pieces of fish containing the medication and were monitored during the entire study to ensure that they did not regurgitate the piece of food or the capsules. In the second study, meloxicam was manually administered using a plastic 10 mm diameter orogastric tube and a 20 ml syringe. The orogastric tube length was estimated for each animal so that the medication was administered directly into the stomach. For this study, the eight individuals were divided into two groups of four. In the first group, meloxicam tablets (Movalis^®^ 7.5 mg/tablet) were crushed to a powder and manually administered in combination with wet dog food (Hill's^®^ a/d prescription diet restorative care wet canned food). In the second group, meloxicam suspension (Metacam^®^ 1.5 mg/ml oral suspension, Boheringer Ingelheim S.A., Barcelona 08174, Spain) was administered with wet dog food using the same orogastric tube. All animals were monitored for regurgitation throughout the study.

**Figure 1:**
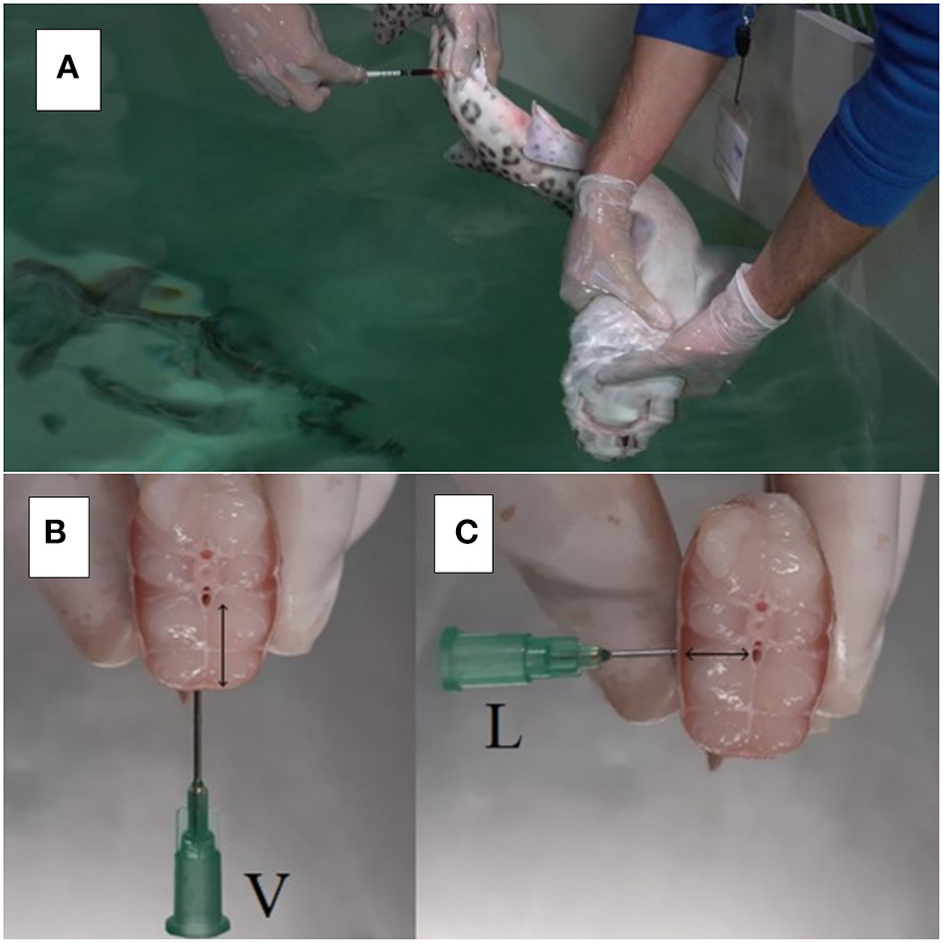
Blood collection in an adult nursehound (*Scyliorhinus stellaris*) during PK study **(A)**. Note that the animal is manually restrained with its head and gills under water and blood is collected via lateral access to the caudal vasculature. Quarantine facilities, Oceanogràfic of Valencia, Spain. Caudal sagittal section in a nursehound shark during necropsy, dorsal is above and ventral is below; detail of the indicated for venipuncture site in elasmobranchs **(B,C)**. Please note that when performing the lateral access (L) to the caudal blood vasculature for venipuncture, the amount of traumatized muscle tissue (↔) is reduced compared to the ventral access (V; ↕).

Blood was drawn using a lateral access of the caudal vasculature with a 25-gauge needle attached to a 1-ml syringe. Each shark was placed in dorsal recumbency to induce TI, allowing efficient blood collection while reducing the possibility of muscular damage. The tail and posterior half of the shark were brought to the surface while the head and gills of the animal were kept underwater (see Figure 1). A volume of 0.4 ml blood was collected before meloxicam administration and at the following times after meloxicam IV administration: 10, 20, 40 min, and 1, 1.5, 2, 4, 6, 9, 12, 24, 36, and 48 h. The same 0.4 ml blood volume was collected in the IM and PO studies, and extraction times after administration were: 15, 30 min, and 1, 3, 6, 9, 12, 24 h. Samples were also collected at 48 h for PO administration. All samples were processed separately.

### Blood Processing

After extraction, blood samples were directly transferred into 1 ml lithium heparin tubes (Aquisel^®^ 1 ml 12x55mm, AQUISEL S.L., Abrera 08630, Spain). Samples were maintained at 4°C, transported to the aquarium's laboratory, and processed within 30 min of their collection. At the laboratory, heparin tubes were centrifuged at 590 g for 5 min at room temperature (24°C) in an Ortoalresa^®^ centrifuge (Ortoalresa^®^ RT106 Na 170007/01, 132 mm rotor radius, 35-degree angle fixed, Ortoalresa-Alvarez Redondo S.A., Daganzo de Arriba 28814, Spain). Plasma was collected and transferred to 1.5 ml Eppendorf tubes, which were then frozen at −20°C and sent to the Pharmacology and Toxicology Department of the Complutense University of Madrid to measure meloxicam concentrations.

### Meloxicam Quantification

Meloxicam concentration in each plasma samples was determined using a previously described reverse-phase high performance liquid chromatographic method, validated for its use in different non-mammalian species (22, 23). In this study, a C18 column (Mediterranean Sea C-18 column; Teknokroma Analítica S.A., Barcelona 08173, Spain) was equipped to the chromatography system (Teknokroma Analítica S.A., Barcelona 08173, Spain). A 20 mM potassium phosphate buffer (pH, 3.5) with acetonitrile (50:50 [vol:vol]) as a mobile phase, which was delivered via an isocratic flow at a rate of 1.2 ml/min. The wavelength of the UV detector was 355 nm. Drug quantification was performed via chromatographic peak integration. For drug concentration measurements, a volume of 0.25 ml shark plasma was mixed with 100 μl of hydrochloride acid solution (5M) and vortexed for 2 min. Afterwards, 3 ml of diethyl ether was added, and the solution was vortexed again for 10 min and centrifuged at 4,500 g and 4°C for 10 min. Finally, the organic layer was collected, transferred to a new test tube, evaporated until dry (at 45°C, under a vacuum stream), and reconstituted in 0.25 ml of methanol for injection into the chromatography system. A calibration curve was created using solutions of known meloxicam concentration (from 1 to 2,500 ng/ml) in methanol, displaying linear absorbance at the studied concentrations (*R*^2^ > 0.99). The limit of detection was 4 ng/ml, the limit of quantification was 15 ng/ml, the inter-assay variability was 5.19% and intra-assay variability was 5.73%. The mean ± SD meloxicam recovery in nursehound plasma samples using this protocol was 92.85 ± 5.30% after adding known concentrations of meloxicam (Sigma-Aldrich Química SA, Tres Cantos 28760, Madrid, Spain) to blank *S.stellaris* plasma.

### Pharmacokinetic Analysis

Mean peak plasma concentration (C_max_) and mean time to peak plasma concentration (T_max_) were calculated directly from the plasma concentrations obtained during the study.

A non-compartmental analysis was performed using a commercially available software (PK Solutions, version 2.0, Summit Research Services, Montrose, Colorado USA) and the following PK parameters were evaluated: plasmatic concentration extrapolated to 0 h (C_0_), elimination half-life (t_1/2β_), area under the plasma concentration computed using observed data points only (AUC_t_), area under the plasma concentration extrapolated to infinity (AUC_inf_), and mean residence time (MRT). AUC after IM and IV administration provide the absolute IM bioavailability (F) of meloxicam. In addition, apparent volume of distribution in pseudo-equilibrium conditions (Vd) and systemic clearance (Cl) were estimated after IV administration and corrected using the F value after IM administration. Mean absorption time (MAT) was calculated as the difference between MRT for IM administration and MRT for IV administration. Plasma concentrations and PK parameters are expressed in this article as means ± SD.

## Results

Meloxicam administered at a dosage of 0.5 mg/kg PO in nursehounds did not produce detectable levels in plasma within 48 h after voluntary or forced administration.

**Figure 2:**
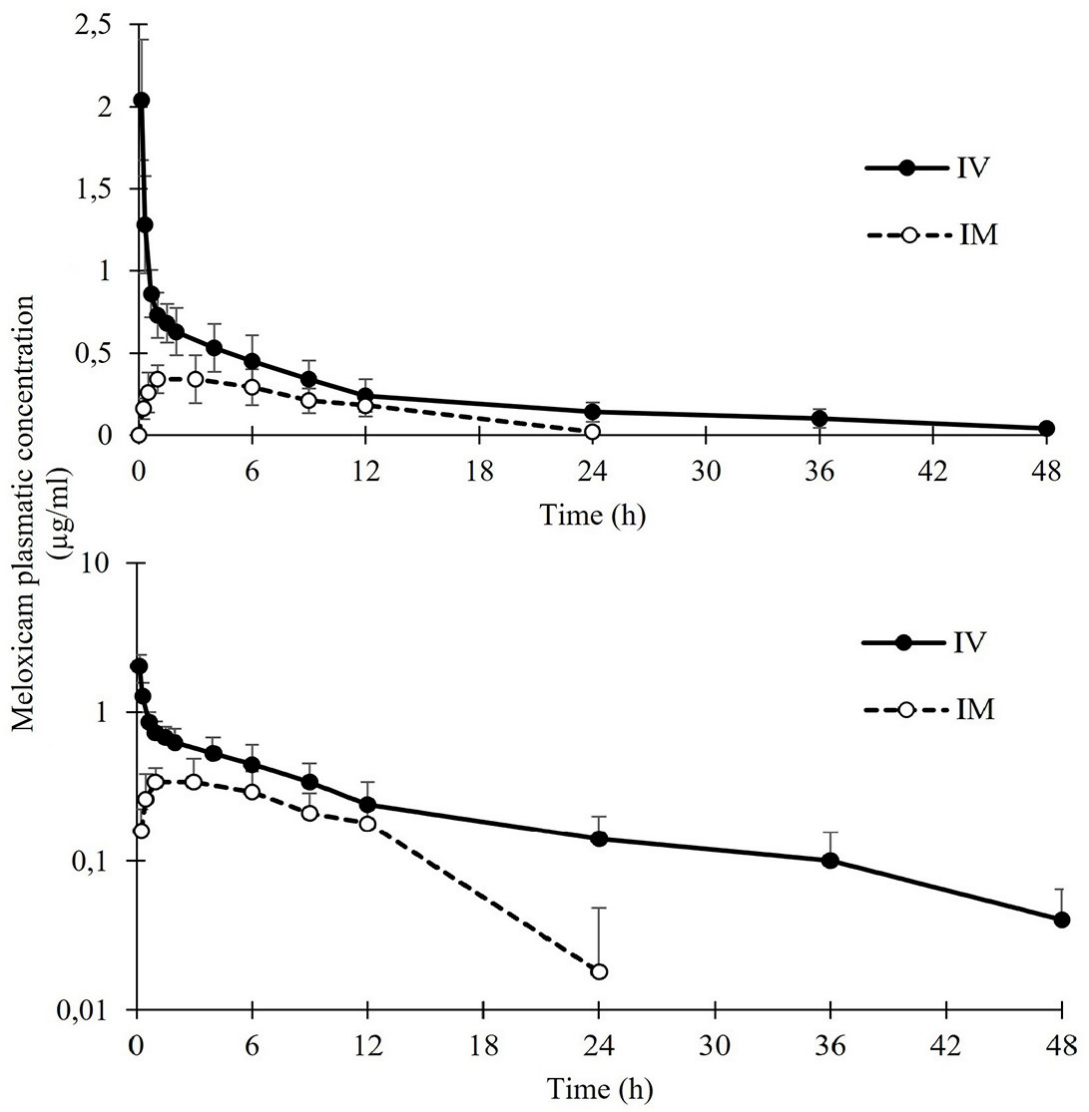
Mean ± SD plasma concentrations of meloxicam in nursehound shark (*Scyliorhinus stellaris*; *n* = 8) after administration of a single IV (solid circles) or IM dose (open circles) (0.5 mg/kg). Note that data are represented using both linear and logarithmic scale.

Mean plasmatic concentrations after IV and IM administrations are represented in Figure 2. PK parameter estimates for 0.5 mg/kg IV and IM meloxicam administration in *S.stellaris* are summarized in Table 1. Meloxicam showed a rapid absorption after IM administration, and elimination was slower and progressive after both IV and IM administrations, with detectable levels in plasma 48 h after IV administration.

**Table 1:** Pharmacokinetic parameters of meloxicam administered at a single dose of 0.5 mg/kg IV or IM in nursehounds (*S.stellaris*; *n* = 8) under human care.

		**IV administration**		**IM administration**	
**Parameter**	**Unit**	**Mean (***n*** = 8)**	**SD**	**Mean (***n*** = 8)**	**SD**
C_0_	μg/ml	3.55	1.30	-	-
T_max_	h	-	-	2.68	1.81
C_max_	μg/ml	2.05	0.22	0.38	0.08
t_1/2β_	h	10.71	2.77	11.27	3.96
AUC_t_	h·μg/ml	10.64	1.95	4.74	1.04
AUC_inf_	h·μg/ml	11.37	2.29	5.98	0.90
MRT	h	15.01	3.45	16.07	5.00
Vd	L/kg	0.67	0.08	0.79	0.31
Cl	ml/min·kg	0.81	0.22	0.81	0.11
F	%	-	-	56.22	13.29
MAT	h	-	-	1.05	0.99

*T_max_, time required to achieve maximum plasmatic concentration; C_0_, extrapolated concentration at 0 h after IV administration; C_max_, maximum plasmatic concentration; t_1/2β_, elimination half-life; AUC_t_, area under the plasma concentration curve computed using observed data points only; AUC_inf_, area under the curve extrapolated to infinity; MRT, mean residence time; Vd, distribution volume in pseudo-equilibrium conditions; Cl, clearance; F, bioavailability; MAT, mean absorption time*.

IV, IM and PO meloxicam administrations were easily performed in nursehounds under the proposed experimental conditions. In the 6 months following the study, there were no shark fatalities or signs of toxicity. Swimming behavior and appetite were also normal in all subjects.

## Discussion

The results provided by this study are relevant for aquatic animal veterinarians and researchers because they present novel data on the PK properties of meloxicam in sharks, following the prevalent administration methods. This study suggests that a correction in the empirical dosages that are recommended and used today in sharks (0.2 mg/kg) may be necessary, due to the low plasma concentrations detected, even when dosages as high as 0.5 mg/kg are administered IM (17). This study uses a dosage of 0.5 mg/kg, rather than 0.2 mg/kg, because a pilot study with 0.5 mg/kg meloxicam IM using two adult nursehounds had already showed relatively low plasma concentrations. Oral meloxicam is still commonly prescribed in elasmobranchs (17). This study shows that neither PO method of administering 0.5 mg/kg meloxicam in nursehounds produces detectable concentrations in plasma. Additionally, this study establishes a safe and efficient experimental model for the development of PK studies using nursehound sharks.

**Figure 3:**
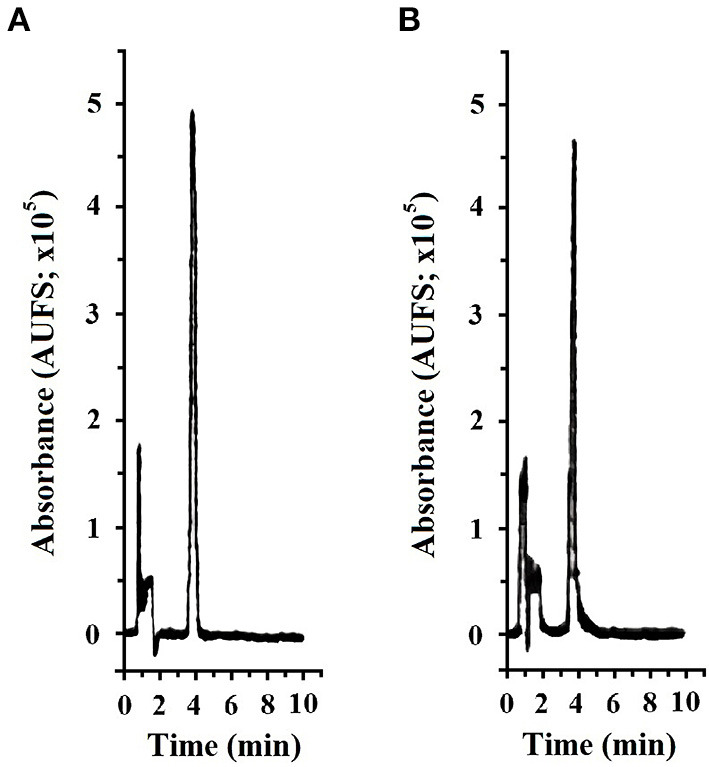
Representative chromatogram (Teknokroma Analítica S.A., Barcelona 08173, Spain) of meloxicam (Sigma-Aldrich Química SA., Tres Cantos 28760, Madrid, Spain) at 1.2 μg/ml concentration in methanol; retention time was 3.91 min and AUC was 132,819 AUFS·min^−1^ **(A)** and in nursehound shark (*Scyliorhinus stellaris*) plasma after processing using the methodology described by Montesinos et al. (22); retention time was 3.85 min and AUC was 111,259 AUFS·min^−1^ **(B)**. Spectra-physics SP4600 Integrator, Lasing S.A., Madrid 28037, Spain.

Nursehounds appear to be a promising candidate species for the further development of PK studies in elasmobranchs, due to their popularity in aquariums and easy handling and management under human care (1). These sharks are one of the largest members in size of the Scyliorhinidae family, while maintaining a relatively small size (max 162 cm total length and 8 kg weight) (21). While handling can be challenging and dangerous in larger elasmobranchs, smaller species may not allow for sufficient collection of blood volumes required to develop a PK study, seeing as the recommended total blood collected from fish should be <1% of its body weight in a 2-week period (24). The small cell fraction present in elasmobranch peripheral blood (mean PCV ranging from 15 to 30%) permits the obtainment of high plasma volumes, reducing the blood volume required to develop the PK analysis (25, 26). The total 5.6 ml, 3.6 ml, and 4.9 ml blood collected over 48 h in the studies performed with IV, IM, PO administration, respectively, represent 0.21, 0.13, and 0.18% of the mean body weight (2.69 kg ± 0.43 kg) of the nursehounds sampled in this study. These values were well below the maximum 1% body weight indicated in fish and shows that the frequent blood sampling required to develop a PK study can be performed in this species while leaving appropriate safety margins (24). Another advantage of nursehounds as subjects is that they are easily induced into TI (14, 27). While some elasmobranch species are less susceptible to TI and can fight and struggle when turned in a dorsal decubitus, muscle relaxation in nursehounds in this study was produced <30 s after dorsal recumbency and allowed for easy blood collection (14). This study collected blood from the indicated site for venipuncture in elasmobranchs, which is the caudal hemal arch. This caudal blood vessel in fish can be accessed via a ventral approach (the most common and intuitive since it is symmetrical) or a lateral approach (more challenging, since it is asymmetrical). As shown in Figure 3, lateral sampling can result in a smaller amount of traumatized muscular tissue and allow for the use of shorter needles in many elasmobranch species. The lateral approach of the caudal hemal arch can be useful in reducing tissue trauma during procedures that require frequent blood sampling, such as PK studies.

Although meloxicam is used frequently in the treatment of inflammatory diseases in elasmobranchs today, there is still very limited information about its PK properties and efficacy in non-domestic animals, particularly in fish (12, 19). A former PK study performed with meloxicam in fish administered at a dosage of 1 mg/kg IM and IV in Nile tilapia (*Oreochromis niloticus*) suggested that meloxicam was not an effective anti-inflammatory drug to be used in fish, due to its extremely short mean elimination half-life (1.8 h) when administered IM (20). A recent PK study administered meloxicam at 1 mg/kg PO in tilapia, obtaining a similar half-life (1.91 h) (28). If we use the PK parameter half-life as an indicator to define possible inter-dosage periods, as it has been suggested in previous PK studies, inter-dosage periods as short as 2 h would not be recommended since frequent capture for IM injection in fish could be counterproductive (20). However, our results show that meloxicam 0.5 mg/kg has a much longer half-life in elasmobranchs (11.27 h) when administered IM, as compared to teleost (29). Furthermore, both mean t_max_ (2.68 vs. 0.50 h) and MRT (16.07 vs. 2.76 h) are longer in nursehounds than in Nile tilapia, supporting the slower absorption, distribution, and elimination of this drug in sharks. These data, together with no local intolerance noted for the drug administered IV or IM, would suggest that IM administration could be an efficient administration route in sharks, though further studies at higher dosages may require longer periods between administrations.

The important differences observed between bony and cartilaginous fish, mainly related to the elimination phase, may be explained by the significant anatomical, physiological, and metabolic differences between the studied species. Meloxicam elimination is mainly hepatic, through conjugation and metabolic reactions via p450 cytochrome; there is also minor renal elimination as a secondary elimination route (30). The size and composition of the liver depend on the species, and in elasmobranchs can represent up to 23% of their body weight and most of it (as high as 80%) can be lipid (31). Moreover, elasmobranchs lack cavitary adipose tissue, storing lipids in hepatocytes; the amount of liver cell exposure, as well as lipids, can influence drug pharmacodynamics (32). The elasmobranch kidney is very different from that of other vertebrates: it has a higher filtration rate as well as different filtration, secretion, and reabsorption selectivity (33). These differences could have influenced the elimination rates of the drug.

Drug leakage at the injection site may explain the decreased AUC_inf_ observed both in teleost and elasmobranchs after IM as compared to IV administration, with a similar F of 53% in nursehounds and 51% in Nile tilapia for IM administration (20). Although manual pressure was applied at the injection site after IM administration, drug leakage has already been described in teleost and elasmobranch fish, due to their different muscle composition, structure and elasticity as compared to mammals (34). This was a limitation to this study, as measuring the amount of drug expelled from the injection site and determining possible ways to reduce leakage should be addressed in future studies. Some possibilities to reduce drug leakage include sealing the injection site after injection, using higher concentration formulas, reducing injection volumes, or distributing the dose across various injection sites. Again, this study aimed to evaluate the current administration protocols for meloxicam in clinical management; additional PK studies would be needed to test the aforementioned hypotheses in elasmobranchs.

Analgesic efficacy of the NSAID ketoprofen at 1–4 mg/kg IM has been studied in the chain dogfish (*Scyliorhinus retifer*), without detecting a significant effect; the study suggested that the lack of appropriate drug doses, administration protocols, and other not yet identified physiological factors could be leading to the lack of apparent analgesic efficacy of this NSAID in sharks (35). In the absence of efficacy studies determining the minimum plasma concentrations of meloxicam necessary for producing analgesia in fish, the effective concentrations determined for other animal species were taken as a reference. Using the established analgesic and anti-inflammatory concentrations, mean meloxicam plasma concentrations ± SD at 12 h after IV and IM administrations (0.239 ± 0.09 μg/ml and 0.181 ± 0.04 μg/ml, respectively) stay above the minimum effective concentrations for ameliorating pain and inflammation in horses (0.130–0.195 μg/ml) (36). However, using effective concentrations in other species like humans (0.57–0.93 μg/ml) or dogs (0.82 μg/ml) as a reference, meloxicam administered IM at 0.5 mg/kg could not reach sufficient plasma concentrations to ameliorate inflammation and pain in sharks (37, 38). These interspecific differences should be interpreted with caution, as the plasma protein binding extent of meloxicam has not been yet determined in fish, and the extrapolation of clinically effective concentrations from other species can lead to an overdose or an insufficient drug administration (39). Future studies determining plasma protein binding of meloxicam in teleost and elasmobranchs are needed to determine the free meloxicam concentration in their plasma. Previous studies conducted with meloxicam administered IM at a dosage of 5 mg/kg in goldfish (*Carassius auratus auratus*) demonstrated that it does not cause acute toxicity in fish (40). This supports the development of future PK trials with higher single-dose and multiple-dose studies, as well as *in vitro* and *in vivo* studies to determine the effective meloxicam concentration for COX-2 inhibition in elasmobranchs. The results from this study indicate that meloxicam should not be used orally at 0.5 mg/kg in nursehounds due to its non-detectable levels in plasma. A recently published study evaluated PK of oral meloxicam in teleost fish for the first time, administering 1 mg/kg meloxicam PO to tilapia, and concluded that oral meloxicam in tilapia would likely not reach clinically effective concentrations because of the significantly low plasmatic concentrations achieved (C_max_ 0.07 μg/ml), with a MRT of 4.11 h and non-detectable concentrations in plasma 8 h after administration (28). Oral administration of meloxicam can, however, be of great use in cases of delicate, easily stressed, or potentially dangerous elasmobranch species since it does not require capture for manual or pole injection. Future trials with higher dosages, delayed release oral formulations, and/or administration with different foods should be conducted to identify possible alternative oral therapeutic regimes (41). Gastrointestinal motility and secretion are the two factors necessary for disintegration and dissolution of solid dosage forms, so differences in these two parameters in teleost and elasmobranchs could have also influenced dissolution and therefore absorption of the drug (42). PK results suggest that oral bioavailability of meloxicam in fish is very limited, and efforts should be taken to better understand why, as oral meloxicam is currently used in elasmobranchs at dosages below those evaluated in this study (17). Furthermore, a hepatic fist-pass effect could have also produced the non-detectable meloxicam levels in plasma after oral administration, rendering oral bioavailability null after meloxicam absorption (43). In all studied species, once meloxicam is absorbed, it undergoes mainly hepatic metabolization, which transform meloxicam into several inactive metabolites (44–46). Plasma and tissue concentrations of meloxicam and its metabolite 5'Hydroxy-desmethyl-meloxicam in tilapia after 1 mg/kg oral administration were all very low, with non-detectable tissue concentrations of its metabolite (28). Another limitation to our study is that the concentration of meloxicam metabolites was not determined in nursehounds, and it is not possible to discard that a hepatic first-pass effect was responsible for the non-detectable levels of meloxicam in plasma after oral administration. Further studies determining plasma and tissue concentrations of meloxicam metabolites in elasmobranchs, including known meloxicam metabolites and possibly still undetermined metabolites in these species, are necessary to understand if this null bioavailability may be caused by a hepatic first-pass effect rather than insufficient drug absorption. However, it should be considered that although the determination of the concentration of meloxicam metabolites would be of great importance to better understand its kinetic properties, since none of its currently determined metabolites are pharmacologically active, the clinical applications of their determination would be limited (46).

Determination of therapeutic regimes following PK studies in elasmobranchs is challenging, since great interspecific variations have been observed in fish administered the same drugs, and elimination has proven temperature-dependent for some active principles (47). Therefore, future studies using higher dosages of meloxicam and multiple administrations are needed. Meloxicam PK properties should also be evaluated in other chondrichthyan species to determine if the elimination rate and half-life is maintained for meloxicam among elasmobranchs and how environmental parameters, such as water temperature and salinity, can influence the PK parameters.

## Conclusions

Meloxicam administered to nursehounds (*Scyliorhinus stellaris*) at a dosage of 0.5 mg/kg presents a more prolonged PK profile when injected IM/IV than in teleost fish. Meloxicam PO did not produce detectable levels in plasma, though plasma concentrations were detectable when the drug was administered IM/IV for at least 12 h, likely resulting in clinically relevant levels. However, in the absence of studies determining the minimum plasma concentrations of meloxicam necessary for effective COX-2 inhibition in fish, the results obtained in this study for IM administration would suggest that 0.5 mg/kg produces stable but low meloxicam plasma levels, which are lower than those associated with anti-inflammatory effects in other animal species. These results suggest that currently recommended dosages in sharks and rays are most likely unable to produce clinically effective plasma concentrations in sharks. They also elucidate the need for further PK studies with higher dosages of meloxicam administered IM in elasmobranchs in order to establish efficient analgesic/anti-inflammatory treatment protocols.

## Data Availability Statement

The raw data supporting the conclusions of this article will be made available by the authors, without undue reservation.

## Ethics Statement

The animal study was reviewed and approved by Animal Care and Welfare Committee at Oceanogràfic of Valencia with the project reference ID OCE-22-19.

## Author Contributions

PM-E: study design, conducting experiments, data acquisition, photography, and preparation of the manuscript. CR-S: conducting experiments, data acquisition, and revision of the manuscript. TÁ-Á and MV-T: conducting experiments and revision of the manuscript. DG-P: study design and revision of the manuscript. TE: study design, data acquisition, and revision of the manuscript. All authors contributed to the article and approved the submitted version.

## Funding

Funding for this study was provided by the Complutense University of Madrid Predoctoral Contract (PME; CT63/19-CT64/19) and the Fundación Oceanogràfic (Under the Project Reference Number OCE-22-19).

## Conflict of Interest

The authors declare that the research was conducted in the absence of any commercial or financial relationships that could be construed as a potential conflict of interest.

## Publisher's Note

All claims expressed in this article are solely those of the authors and do not necessarily represent those of their affiliated organizations, or those of the publisher, the editors and the reviewers. Any product that may be evaluated in this article, or claim that may be made by its manufacturer, is not guaranteed or endorsed by the publisher.
