# Erratum: Pharmacokinetic studies in elasmobranchs: Meloxicam administered at 0.5 mg/kg using intravenous, intramuscular, and oral routes to nursehound sharks (*Scyliorhinus stellaris*)

**DOI:** 10.3389/fvets.2023.1136968

**Published:** 2023-01-18

**Authors:** Frontiers Production Office

**Affiliations:** Frontiers Media SA, Lausanne, Switzerland

**Keywords:** meloxicam, shark, chondrichthyan, pharmacology, pharmacokinetics, non-steroidal anti-inflammatory drug (NSAID), half-life (T1/2)

Due to a production error, there was a spelling error in the title “Pharmacokinetic Studies in Elasmobranchs: Meloxicam Administered at 0.5 mg/kg Using Intravenous, Intramuscular, and Oral Routes to Nusehound Sharks (*Scyliorhinus stellaris*)”. The correct title is “Pharmacokinetic Studies in Elasmobranchs: Meloxicam Administered at 0.5 mg/kg Using Intravenous, Intramuscular, and Oral Routes to Nursehound Sharks (*Scyliorhinus stellaris*)”.

The publisher apologizes for this mistake. The original version of this article has been updated.

